# First-Line Antituberculosis Drug Concentrations in Infants With HIV and a History of Recent Admission With Severe Pneumonia

**DOI:** 10.1093/jpids/piad088

**Published:** 2023-10-16

**Authors:** Chishala Chabala, Tom G Jacobs, Cinta Moraleda, John M Ndaferankhande, Vivian Mumbiro, Alfeu Passanduca, Natasha Namuziya, Damalie Nalwanga, Victor Musiime, Alvaro Ballesteros, Sara Domínguez-Rodríguez, Moses Chitsamatanga, Uneisse Cassia, Bwendo Nduna, Justina Bramugy, Jahit Sacarlal, Lola Madrid, Kusum J Nathoo, Angela Colbers, David M Burger, Veronica Mulenga, W Chris Buck, Hilda A Mujuru, Lindsey H M te Brake, Pablo Rojo, Alfredo Tagarro, Rob E Aarnoutse, EMPIRICAL clinical trial group, Muhammad Sidat, Elias Manjate, Sónia Martins, Stella Langa, Natália Nipaco, Sara Machava, Anastância Chirindza, Luzidina Martins, Mércia Nhaca, Kusum J Nathoo, Moses Chitsamatanga, Ruth Marange, Shepherd Mudzingwa, Dorothy Murungu, Idah Zulu, Perfect Shankalala, Mulima Mukubesa, Juliet Namwinwa, Chalwe Chibuye, Terence Chipoya, Bwalya Simunyola, John Tembo, Muleya Inambao, Salome Chitondo, Wyclef Mumba, Endreen Mankushe, Henry Musukwa, Davies Sondashi, Albert Kamugisha, Karen Econi, Andrew Kiggwe, Judith Beinomugisha, Sharafat Nkinzi, Lawrence Kakooza, Henriator Namisanvu, Nancy Lajara Mark, Josam Thembo Mwesige, Ivan Segawa, Joseph Ssessanga, Paul Mbavu, Bosco Kafufu, Denis Nansera, Elizabeth Najjingo, Bashira T Mbabazi, Abbas Lugemwa, Mariam Kasozi, Rogers Ankunda, Lilit Manukyan

**Affiliations:** University of Zambia, School of Medicine, Lusaka, Zambia; University Teaching Hospital, Children’s Hospital, Lusaka, Zambia; HerpeZ, Lusaka, Zambia; Department of Pharmacy, Radboudumc Institute for Medical Innovation (RIMI), Radboud University Medical Center, Nijmegen, The Netherlands; Pediatric Unit for Research and Clinical Trials (UPIC), Hospital 12 de Octubre Health Research Institute (i+12), Biomedical Foundation of Hospital Universitario 12 de Octubre (FIB-H12O), Madrid, Spain; Malawi-Liverpool-Wellcome Trust Clinical Research Programme, Kamuzu University of Health Sciences, Blantyre, Malawi; University of Zimbabwe Clinical Research Centre, Harare, Zimbabwe; Universidade Eduardo Mondlane, Faculdade de Medicina, Maputo, Mozambique; University Teaching Hospital, Children’s Hospital, Lusaka, Zambia; Department of Paediatrics and Child Health, School of Medicine, College of Health Sciences, Makerere University, Kampala, Uganda; Department of Paediatrics and Child Health, School of Medicine, College of Health Sciences, Makerere University, Kampala, Uganda; Joint Clinical Research Centre, Kampala, Uganda; Pediatric Unit for Research and Clinical Trials (UPIC), Hospital 12 de Octubre Health Research Institute (i+12), Biomedical Foundation of Hospital Universitario 12 de Octubre (FIB-H12O), Madrid, Spain; Pediatric Unit for Research and Clinical Trials (UPIC), Hospital 12 de Octubre Health Research Institute (i+12), Biomedical Foundation of Hospital Universitario 12 de Octubre (FIB-H12O), Madrid, Spain; University of Zimbabwe Clinical Research Centre, Harare, Zimbabwe; Universidade Eduardo Mondlane, Faculdade de Medicina, Maputo, Mozambique; Arthur Davidson Children’s Hospital, Ndola, Zambia; Centro de Investigação em Saúde de Manhiça, Maputo, Mozambique; Universidade Eduardo Mondlane, Faculdade de Medicina, Maputo, Mozambique; Pediatric Unit for Research and Clinical Trials (UPIC), Hospital 12 de Octubre Health Research Institute (i+12), Biomedical Foundation of Hospital Universitario 12 de Octubre (FIB-H12O), Madrid, Spain; London School of Hygiene and Tropical Medicine (LMC), London, UK; University of Zimbabwe Clinical Research Centre, Harare, Zimbabwe; Department of Pharmacy, Radboudumc Institute for Medical Innovation (RIMI), Radboud University Medical Center, Nijmegen, The Netherlands; Department of Pharmacy, Radboudumc Institute for Medical Innovation (RIMI), Radboud University Medical Center, Nijmegen, The Netherlands; University of Zambia, School of Medicine, Lusaka, Zambia; University Teaching Hospital, Children’s Hospital, Lusaka, Zambia; Universidade Eduardo Mondlane, Faculdade de Medicina, Maputo, Mozambique; University of California Los Angeles, David Geffen School of Medicine, Los Angeles, California, USA; University of Zimbabwe Clinical Research Centre, Harare, Zimbabwe; Department of Pharmacy, Radboudumc Institute for Medical Innovation (RIMI), Radboud University Medical Center, Nijmegen, The Netherlands; Pediatric Unit for Research and Clinical Trials (UPIC), Hospital 12 de Octubre Health Research Institute (i+12), Biomedical Foundation of Hospital Universitario 12 de Octubre (FIB-H12O), Madrid, Spain; Complutense University of Madrid, Madrid, Spain; Pediatric Service, Hospital Universitario 12 de Octubre, Servicio Madrileño de Salud (SERMAS), Madrid, Spain; Pediatric Unit for Research and Clinical Trials (UPIC), Hospital 12 de Octubre Health Research Institute (i+12), Biomedical Foundation of Hospital Universitario 12 de Octubre (FIB-H12O), Madrid, Spain; Pediatric Service, Infanta Sofia University Hospital, Servicio Madrileño de Salud (SERMAS), Madrid, Spain; Universidad Europea de Madrid, Madrid, Spain; Department of Pharmacy, Radboudumc Institute for Medical Innovation (RIMI), Radboud University Medical Center, Nijmegen, The Netherlands

**Keywords:** HRZE, Tuberculosis, HIV, pharmacokinetics, infants

## Abstract

Optimal antituberculosis therapy is essential for favorable clinical outcomes. Peak plasma concentrations of first-line antituberculosis drugs in infants with living HIV receiving WHO-recommended dosing were low compared with reference values for adults, supporting studies on increased doses of first-line TB drugs in infants.

## Teaser

First-line antituberculosis drug peak concentrations in infants with HIV were low compared with reference values for adults. The percent peak concentrations within adult reference values were 51%, 22%, 76%, and 6% for isoniazid, rifampicin, pyrazinamide, and ethambutol, respectively.

## INTRODUCTION

Tuberculosis (TB) is a leading cause of death in children living with HIV. Of the 214 000 TB deaths among people living with HIV in 2021, children accounted for 10% [1]. Infants are at risk of developing severe forms of TB when compared with older children [1, 2]. Optimal TB treatment is crucial to avert TB-associated mortality in infants with HIV.

Due to dynamic alterations in metabolic capacity and body composition during growth, infants are susceptible to changes in the pharmacokinetics of drugs that in turn impact the determination of appropriate doses [3]. Pharmacokinetic studies show that children weighing <8 kg, who are dosed according to current WHO-recommended weight bands using fixed-dose combination (FDC) drugs, have lower plasma exposures of first-line TB drugs than adults [4, 5]. Furthermore, the presence of HIV infection is associated with lower exposures to rifampicin and ethambutol [6, 7]. In addition, infants living with HIV and TB are also highly vulnerable to opportunistic infections, and severe acute malnutrition that necessitate multidrug treatment with an associated risk of drug–drug interactions [8]. Antimycobacterial activity and treatment response observed in adults treated for TB are closely linked to plasma or serum drug exposures [9]. In the absence of target drug exposures for children, it is generally agreed that pediatric doses of TB drugs should result in similar exposures to those in adults [7].

Limited pharmacokinetic data of TB drugs are available for low-weight infants with HIV, particularly for infants weighing less than 4 kg [7]. We aimed to evaluate plasma concentrations of first-line TB drugs in infants below 1 year of age living with HIV who were hospitalized for severe pneumonia and received TB treatment during the EMPIRICAL trial.

## METHOD

### Study Population and Design

This was a single-arm pharmacokinetic substudy within the EMPIRICAL multicenter, open-label randomized controlled clinical trial (#NCT03915366) funded by EDCTP which aimed to assess the efficacy of empirical treatment with first-line TB treatment and/or valganciclovir for infants living with HIV who were admitted with severe pneumonia [8]. The main trial enrolled infants between 28 and 365 days old with confirmed HIV infection and severe pneumonia. All eligible infants received standard of care, including antibiotics, cotrimoxazole treatment with prednisolone, and antiretroviral treatment. They were randomized to receive no additional treatment, first-line TB treatment, valganciclovir for 15 days, or both (4 arms) [8]. Infants who received TB treatment either as part of trial randomization or who were diagnosed with TB post-randomization were enrolled from hospitals in Mozambique, Uganda, Zambia, and Zimbabwe.

### Procedures

Pediatric isoniazid, rifampicin, and pyrazinamide FDC dispersible tablets (50/75/150 mg), ethambutol 100 mg dispersible tablets (intensive phase of TB treatment) and isoniazid and rifampicin FDC tablets (50/75 mg, continuation phase, all WHO-prequalified and from manufacturer Macleods) were administered once-daily according to WHO weight-band dosing [10]. The dosages are included in Supplementary File 1. A single blood sample was taken at 2 hours after dose administration (*C*_2h_) at day 30, 90, and 180 after enrollment in the main trial. Samples drawn outside the 1.5–2.5-hour timeframe after drug administration were excluded from the analysis. Food intake around dose administration and adherence 3 days prior to the pharmacokinetic visit were recorded. Isoniazid, acetyl-isoniazid, rifampicin, pyrazinamide, and ethambutol concentrations were determined using a validated liquid chromatography-tandem mass spectrometry (LC-MS/MS) method, as described in Supplementary File 2.

### Statistical Analysis

The population geometric mean (GM, %coefficient of variation) *C*_2h_ was determined for all drugs. We considered *C*_2h_ to be a surrogate parameter for *C*_max_, and hence compared it to reference *C*_max_ values. To compare with a similar population, although predominantly HIV-negative, we used *C*_max_ data for children in the 4–7.9 kg weight band reported in an earlier intensive pharmacokinetic (PK) sampling study [5]. Additionally, the percentages of infants within the adult reference *C*_max_ values were reported [9]. Spearman rank correlation and the Mann–Whitney *U*-test were used to assess bivariate associations between continuous (dose/kg, height, weight-for-length z-score [WLZ], weight-for-age z-score, age, and estimated glomerular filtration rate) and categorical (sex and acetylator status) covariates, respectively, and *C*_2h_ values of TB drugs. Then, for covariates correlating with *C*_2h_ at a significance level of *P* < .1, multiple linear regression on log-transformed data was conducted to test if associations held true after correcting for other covariates.

### Ethics

The study protocol was approved by Investigation Ethics Committee of Medicines Hospital 12 de Octubre in Spain (#19/096) and by local ethics committees. The study consent documents were translated into local languages and all caregivers provided written informed consent.

## RESULTS

### Population

Forty-nine of 50 infants enrolled were included in the analysis (Table 1). One infant was excluded as the blood sample was drawn outside of the 1.5–2.5 hour range after dosing. All children were fed within 2 hours prior to or after dose administration.

**Table 1.** Patient characteristics and pharmacokinetic results on study visit day 30 from all included infants (*n* = 49)

Characteristic (study visit day 30; *n* = 49)	Value	
Sex (*n*)		
Female	21 (43%)	
Male	28 (57%)	
Median (IQR) age (months)	5.6 (4.4 to 9.1)	
Median (IQR) weight (kg)	5.3 (4.8 to 6.2)	
Median (IQR) length (cm)	61 (58 to 65)	
Weight bands (*n*)		
<4.0 kg	5 (10%)	
4.0–7.9 kg	41 (84%)	
8.0–11.9 kg	3 (6%)	
Median (IQR) WLZ	−1.80 (−2.60 to −0.35)	
Median (IQR) WAZ	−2.60 (−4.05 to −1.70)	
Median (IQR) eGFR^a^ (mL/min/1.73 m^2^)	105 (81 to 166)	
Acetylator status (*n*)^b^		
Slow	33 (67%)	
Intermediate/fast	16 (33%)	
Confirmed TB (*n*)^c^		
Yes	8 (16%)	
No	41 (84%)	
Median (IQR) drug dose (mg/kg)		
Isoniazid	9.6 (8.2 to 11.1)	
Rifampicin	14.4 (12.3 to 16.7)	
Pyrazinamide	28.8 (24.6 to 33.7)	
Ethambutol	19.2 (16.4 to 22.5)	
ART regimen during PK sampling visit (*n*)^d^		
DTG-based	10 (21%)	
EFV-based	1 (2%)	
LPVr-based	12 (24%)	
NVP-based	2 (4%)	
Triple NRTI	16 (33%)	
None	8 (16%)	

PK parameters (study visit day 30)		References
Geometric mean (mg/L; %CV) *C*_2h_		Infant references^e^
Isoniazid	2.8 (102)	4.0 (2.3–4.7)
Rifampicin	3.7 (161)	4.9 (3.9–6.4)
Pyrazinamide	22.3 (97)	25.8 (22.3–31.8)
Ethambutol	0.56 (101)	1.2 (0.9–1.6)
Percentage of infants with *C*_2h_ within adult *C*_max_ references		*Adult reference*s^f^
Isoniazid	51%	3–6 mg/L
Rifampicin	22%	8–24 mg/L
Pyrazinamide	76%	20–60 mg/L
Ethambutol	6%	2–6 mg/L

^a^eGFR was calculated using the Schwartz equation for children under 1 year old: eGFR = 0.44 × length (cm)/creatinine (mg/dL).

^b^Phenotypic INH acetylator status was determined by calculating the metabolic ratio for *C*_2h_ (acetyl-INH/INH). Infants having a metabolic ratio below 0.73 were considered slow acetylators and those with higher ratios intermediate/fast acetylators [11].

^c^Diagnosed by GeneXpert and/or TB urine lipoarabinomannan (TB-LAM).

^d^Eight infants were ART naïve during the pharmacokinetic sampling visit. These were very sick infants at the time of recruitment and ART initiation was delayed due to the risk of IRIS (*n* = 4) or due to elevated liver enzymes (*n* = 4).

^e^These reference values represent the median (IQR) *C*_max_ for 16 infants weighing 4–7.9 kg that received drug dosages similar to our population reported by Chabala et al [5]. The majority of these infants (75%) ware HIV-negative.

^f^These reference values represent the normal *C*_max_ that can be expected in adults after the standard doses of TB drugs. They are based on data that were compiled from all available sources (both healthy volunteers and TB patients) [9].

Abbreviations: ART, antiretroviral treatment; CV, coefficient of variation; DTG, dolutegravir; EFV, efavirenz; eGFR, estimated glomerular filtration rate; IQR, interquartile range; LPVr, ritonavir-boosted lopinavir; NRTI; nucleoside reverse transcriptase inhibitor; NVP, nevirapine; PK, pharmacokinetic; TB, tuberculosis; WAZ, weight-for-age z-score; WLZ, weight-for-length z-score.

### Pharmacokinetics

**Figure 1.**
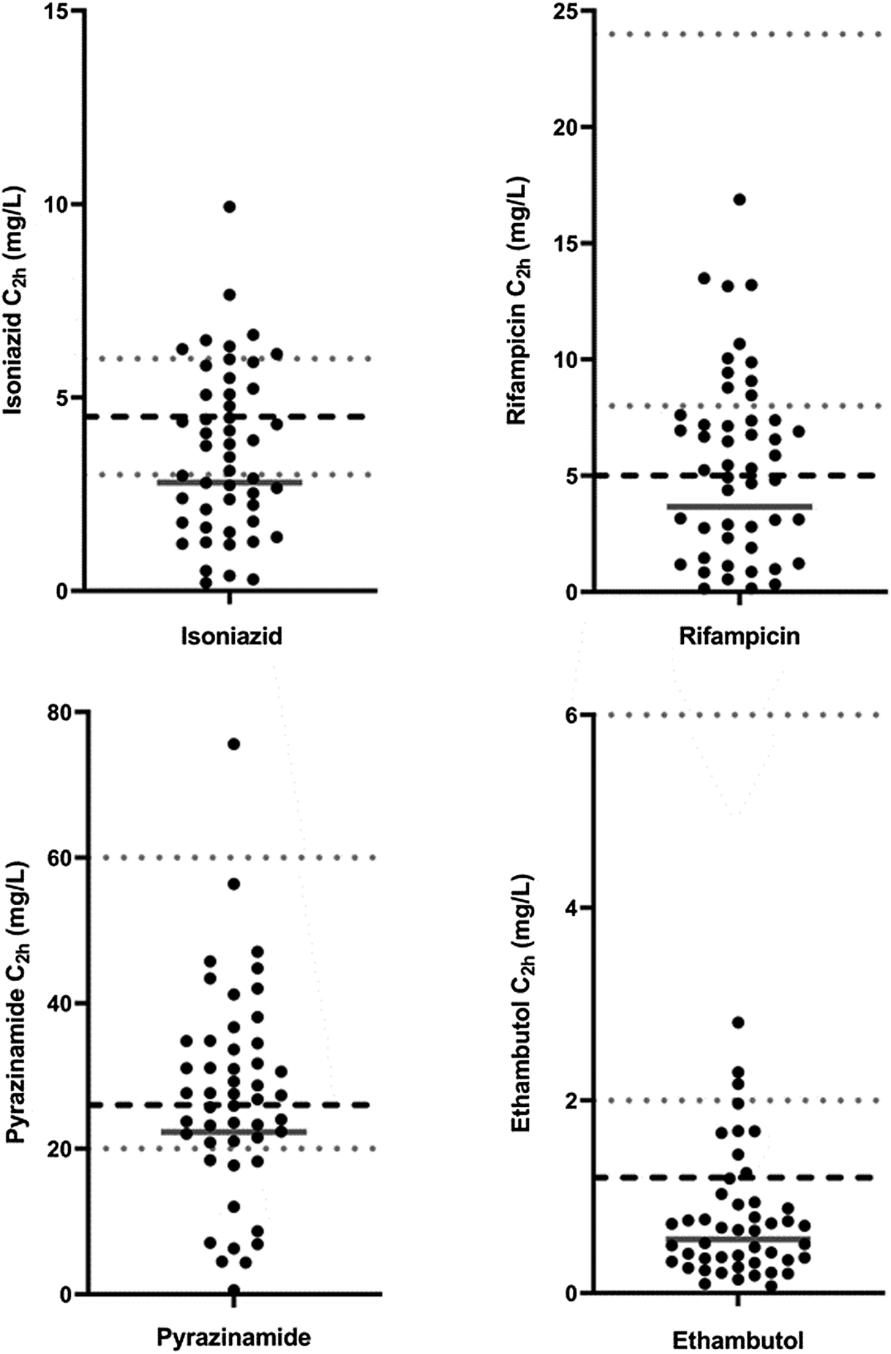
Individual TB drug concentrations on study visit day 30 at 2 hours after dose administration (C_2h_). The grey solid lines represent the geometric means of the individual C_2h_, the grey dotted lines represent the adult target C_max_ ranges as reported by Alsultan et al. [9], and the black dashed lines represent the median C_max_ for children within the 4-7.9 kg weight-band as reported by Chabala et al. [5]. Top left panel: isoniazid; top right panel: rifampicin; bottom left panel: pyrazinamide; bottom right panel: ethambutol.

Table 1 displays GM *C*_2h_ values and individual *C*_2h_ for isoniazid, rifampicin, pyrazinamide, and ethambutol are displayed in Figure 1, highlighting the large interpatient variability in exposure to each of the drugs. Individual *C*_2h_ levels seemed comparable across the various weight bands (<4.0 kg; 4.0–7.9 kg; 8.0–11.9 kg) for all compounds, as displayed in Supplementary File 3. However, only few children were included in the <4.0 kg (*n* = 5) and 8.0–11.9 kg (*n* = 3) weight bands.

The GM isoniazid *C*_2h_ was 2.80 (102) mg/L at day 30 of the trial, with 51% of infants having a *C*_2h_ within the adult *C*_max_ reference range of 3–6 mg/L. Isoniazid *C*_2h_ did not statistically differ per acetylator group, see Supplementary Files 5 and 6. Furthermore, isoniazid and rifampicin concentrations at visit days +90 and +180 were comparable to day +30 (Supplementary File 4). For rifampicin, the GM *C*_2h_ was 3.7 (161) mg/L, with only 22% of infants falling within the adult reference range of 8–24 mg/L. Pyrazinamide GM *C*_2h_ was 22.3 (97) mg/L, with 76% of infants within the adult reference range of 20–60 mg/L. Two infants had undetectable ethambutol concentrations after supervised drug intake and hence were excluded from the analysis. The GM ethambutol *C*_2h_ was 0.56 (101) mg/L, with only 6% of infants within the adult reference range of 2–6 mg/L.

In the multivariable analysis, increasing WLZ was significantly but weakly associated with decreased rifampicin *C*_2h_ and ethambutol concentrations were higher in females, details of the analyses are included in Supplementary File 6. None of the covariates showed a statistically significant effect on isoniazid and pyrazinamide *C*_2h_ in the multivariable analysis.

## DISCUSSION AND CONCLUSION

We found that GM *C*_2h_ of rifampicin, isoniazid, and ethambutol values in infants living with HIV receiving TB treatment following WHO weight-band dosing were below the adult reference *C*_max_ values, whereas the GM *C*_2h_ of pyrazinamide fell within the lower end of the wide adult reference range. The findings were consistent over study visit day 30 after TB treatment initiation and 90, and 180 for the 2 drugs administered beyond 2 months, isoniazid and rifampicin. A dose corresponding with the 4–7.9 kg weight band provided appropriate TB drug exposure in 4 children weighing <4 kg. Of note, there is no formal dose recommendation for FDC tablets for children weighing <4 kg. An alarmingly high proportion of infants had a *C*_2h_ below the adult reference window for rifampicin (78%) and ethambutol (94%). These findings are consistent with other studies that reported low *C*_2h_ for first-line TB drugs in infants weighing <8 kg and children living with HIV, using the current WHO weight-band dosing [5–7].

Previous studies reported malnutrition to be associated with lower total TB drug levels [7]. Many (47%) infants in our study were malnourished, which may have contributed to the low *C*_2h_ in our population. Conversely, we found a significant association between increased rifampicin *C*_2h_ in children and low-WLZ in the multivariable analysis [7]. Of note, weight-for-length measurements in infants are challenging and an appropriate WLZ reference standard for infants below 6 months is less accurate, whilst 53% of our population was under 6 months old. Furthermore, we did not find a significant difference in isoniazid *C*_2h_ for infants with different acetylator status. This may be explained by a more pronounced effect of acetylator status on isoniazid area under curve (AUC) compared with *C*_max_ [11].

This study has several limitations. First, to limit the volume of blood to be drawn from the infants in the study, we drew a single PK sample and hence cannot provide full pharmacokinetic profiles. Secondly, while the *C*_2h_ timepoint approximates *C*_max_, we may not have captured the *C*_max_ for some children due to interpatient pharmacokinetic variability. Additionally, the actual time to *C*_max_ (*T*_max_) for each drug may vary, with isoniazid and pyrazinamide often having a *T*_max_ of slightly less than 2 hours in children while that of rifampicin and ethambutol is often slightly higher than 2 hours. Interpretation of *C*_2h_ may further be complicated because of the fed state of our populations, which could result in delayed drug absorption [12]. Low *C*_2h_ levels do not rule out the possibility of delayed absorption, regardless of fed state. Moreover, *C*_max_ values are generally lower in patients who are fed compared with those who are fasted and may not correlate well with the total exposure (AUC) to the medications. To gather comprehensive data while minimizing the burden on infants, future studies in this population should consider employing limited-sampling strategies to predict AUC values or collecting single pharmacokinetic samples at different timepoints at each study visit to facilitate AUC prediction through population pharmacokinetic modeling. Lastly, we were unable to determine the relevance of the low drug concentrations in terms of efficacy, as the main trial efficacy outcomes were still blinded and confidential at the time of this analysis.

Our findings confirm low plasma concentrations of first-line TB drugs in a vulnerable population of infants with advanced HIV and a history of recent admission with severe pneumonia and were malnourished. These data support large clinical studies investigating increased doses of the first-line TB drugs in FDC and loose ethambutol in infants with HIV.

## Supplementary Data

Supplementary materials are available at the *Journal of The Pediatric Infectious Diseases Society* online (http://jpids.oxfordjournals.org).

EMPIRICAL clinical trial group:

Muhammad Sidat, Elias Manjate, Sónia Martins (Universidade Eduardo Mondlane Faculdade de Medicina, Maputo, Mozambique); Stella Langa, Natália Nipaco (Hospital Central de Maputo, Maputo, Mozambique); Sara Machava, Anastância Chirindza (Hospital Provincial de Matola, Matola, Mozambique); Luzidina Martins, Mércia Nhaca (Hospital Geral de Mavalane, Maputo, Mozambique); Dalila Rego, Dália Machel (Hospital Geral José Macamo, Maputo, Mozambique); Amir Seni, Kajal Chhanganlal, Belinda Macmillan, Aurora Mucarenga, Adelina Manheche (Hospital Central da Beira, Beira, Mozambique); Kusum J Nathoo, Moses Chitsamatanga, Ruth Marange, Shepherd Mudzingwa, Dorothy Murungu (University of Zimbabwe Clinical Research Centre); Idah Zulu, Perfect Shankalala, Mulima Mukubesa, Juliet Namwinwa, Chalwe Chibuye, Terence Chipoya, Bwalya Simunyola, John Tembo (University Teaching Hospital, Lusaka, Zambia); Muleya Inambao, Salome Chitondo, Wyclef Mumba, Endreen Mankushe, Henry Musukwa, Davies Sondashi (Arthur Davidson Children’s Hospital, Ndola, Zambia); Albert Kamugisha, Karen Econi (China Uganda Friendship Hospital Naguru, Kampala, Uganda); Andrew Kiggwe, Judith Beinomugisha, Sharafat Nkinzi, Lawrence Kakooza, Henriator Namisanvu, Nancy Lajara Mark, Josam Thembo Mwesige, Ivan Segawa, Joseph Ssessanga, Paul Mbavu (Makerere University Lung Institute, Kampala, Uganda); Bosco Kafufu (Infectious Diseases Institute Laboratory, Makerere University, Kampala, Uganda); Denis Nansera, Elizabeth Najjingo, Bashira T Mbabazi (Mbarara Regional Referral Hospital, Mbarara, Uganda); Abbas Lugemwa, Mariam Kasozi, Rogers Ankunda (Joint Clinical Research Centre, Regional Centre of Excellence, Mbarara, Uganda); Lilit Manukyan (Pediatric Unit for Research and Clinical Trials (UPIC), Hospital 12 de Octubre Health Research Institute (i + 12), Biomedical Foundation of Hospital Universitario 12 de Octubre (FIB-H12O), Madrid, Spain).

piad088_suppl_Supplementary_Files_1-6

## Data Availability

The data that support the findings of this study are available from the corresponding authors upon reasonable request.

## Notes

***Financial support***. This project is part of the EDCTP2 programme supported by the European Union RIA2017MC-2013.

***Potential conflicts of interest***. The authors declare that they have no conflict of interest.
