# Supplementary material for: First-Line Antituberculosis Drug Concentrations in Infants With HIV and a History of Recent Admission With Severe Pneumonia
Source: J Pediatric Infect Dis Soc. 2023 Oct 16;12(11):581–5. doi: 10.1093/jpids/piad088 (PMC10687595; doi:10.1093/jpids/piad088)
Supplement: piad088_suppl_Supplementary_Files_1-6 [file piad088_suppl_supplementary_files_1-6.docx]

# Supplementary files *First-line antituberculosis drug concentrations in infants with HIV and a history of recent admission with severe pneumonia*

Supplementary file 1 – Dosing table for TB drug dispersible tablets

Supplementary file 2 – Bioanalysis methodology

Supplementary file 3 – Individual C2h levels aggregated per weight-band

Supplementary file 4 – Isoniazid and rifampicin C2h at various study visits

Supplementary file 5 – Isoniazid C2h aggregated per phenotypic acetylator status

Supplementary file 6 – Results covariate analyses

## Supplementary file 1 – Dosing table for TB drug dispersible tablets

| Weight-band | Numbers of tablets | | | |
| --- | --- | --- | --- | --- |
|  | **Intensive phase**  **RHZ 75/50/150** | **Intensive phase**  **EMB 100** | **Continuation phase**  **RH 75/50** |  |
| <4 kg* | 0.5 | 0.5 | 0.5 |  |
| 4-7 kg | 1 | 1 | 1 |  |
| 8-11 kg | 2 | 2 | 2 |  |

**Table 1 Provided dosages of isoniazid, rifampicin, pyrazinamide, and ethambutol per weight-band in the study.**
* There is no recommendation for this weigh band in WHO guidelines. Infants below 4 kg should have received half FDC and half ethambutol dispersible tablet. The safety and efficacy of this formulation have not been assessed in large studies in infants, but equivalent liquid formulations are used routinely. In this study, 4 children weighing less than 4kg were included of whom one received half tablets while the other three received a full dispersible tablet.
Abbreviations: EMB, ethambutol; RH, rifampicin+isoniazid; RHZ, rifampicin+isoniazid+pyrazinamide.

## Supplementary file 2 – Bioanalysis methodology

Samples were immediately put on wet ice after sampling and processed within 1 hour, meeting the stability requirements for isoniazid. Plasma was then stored at -80^o^C at each clinical site before shipment on dry ice to the Department of Pharmacy, Radboud university medical center, Nijmegen, The Netherlands. A material transfer agreement was signed between the laboratory, the sponsor, and all the participating sites separately, and was approved by relevant authorities in all participating countries. Drug concentrations were quantified using a liquid chromatography with tandem mass spectrometry (LC-MS/MS) assay. The assay was validated according to the most recent European Medicines Agency (EMA) guidelines for bioanalytical method validation and the laboratory participates in an international interlaboratory quality control program for bioanalysis of anti-tuberculosis drugs. Table 2 presents the detection range for all compounds, the within-run and between-run precision reported as coefficient of variation, and the accuracy.

|  | | Conc. | Within run (n=5) | | Between run (n=15) | |
| --- | --- | --- | --- | --- | --- | --- |
|  |  |  | *Accuracy* | *Precision* | *Accuracy* | *Precision* |
|  |  | *(mg/L)* | *(%)* | *(%)* | *(%)* | *(%)* |
| INH | LLOQ | 0.045 | 94.2 | 5.4 | 94.8 | 0.0 |
|  | HLOQ | 15.1 | 104.1 | 6.1 | 103.1 | 0.0 |
| Ac-INH | LLOQ | 0.045 | 98.0 | 4.4 | 99.5 | 0.0 |
|  | HLOQ | 15.0 | 95.7 | 4.3 | 97.7 | 0.8 |
| RIF | LLOQ | 0.091 | 95.9 | 4.9 | 97.9 | 1.9 |
|  | HLOQ | 60.8 | 97.6 | 2.7 | 98.0 | 0.0 |
| PZA | LLOQ | 0.182 | 110.0 | 9.9 | 100.5 | 7.6 |
|  | HLOQ | 60.7 | 97.8 | 8.2 | 99.0 | 0.0 |
| EMB | LLOQ | 0.046 | 93.1 | 4.8 | 96.4 | 2.4 |
|  | HLOQ | 15.2 | 97.2 | 0.9 | 98.0 | 0.8 |

**Table 2. Detection limit and accuracy and precision of the bioanalytical assays.** All values represent mg/L concentrations.
Abbreviations: Ac-INH, acetyl-isoniazid; EMB, ethambutol; HLOQ, higher limit of quantification; INH, isoniazid; LLOQ, lower limit of quantification; PZA, pyrazinamide; RIF, rifampicin

## Supplementary file 3 – Individual C_2h_ levels aggregated per weight-band


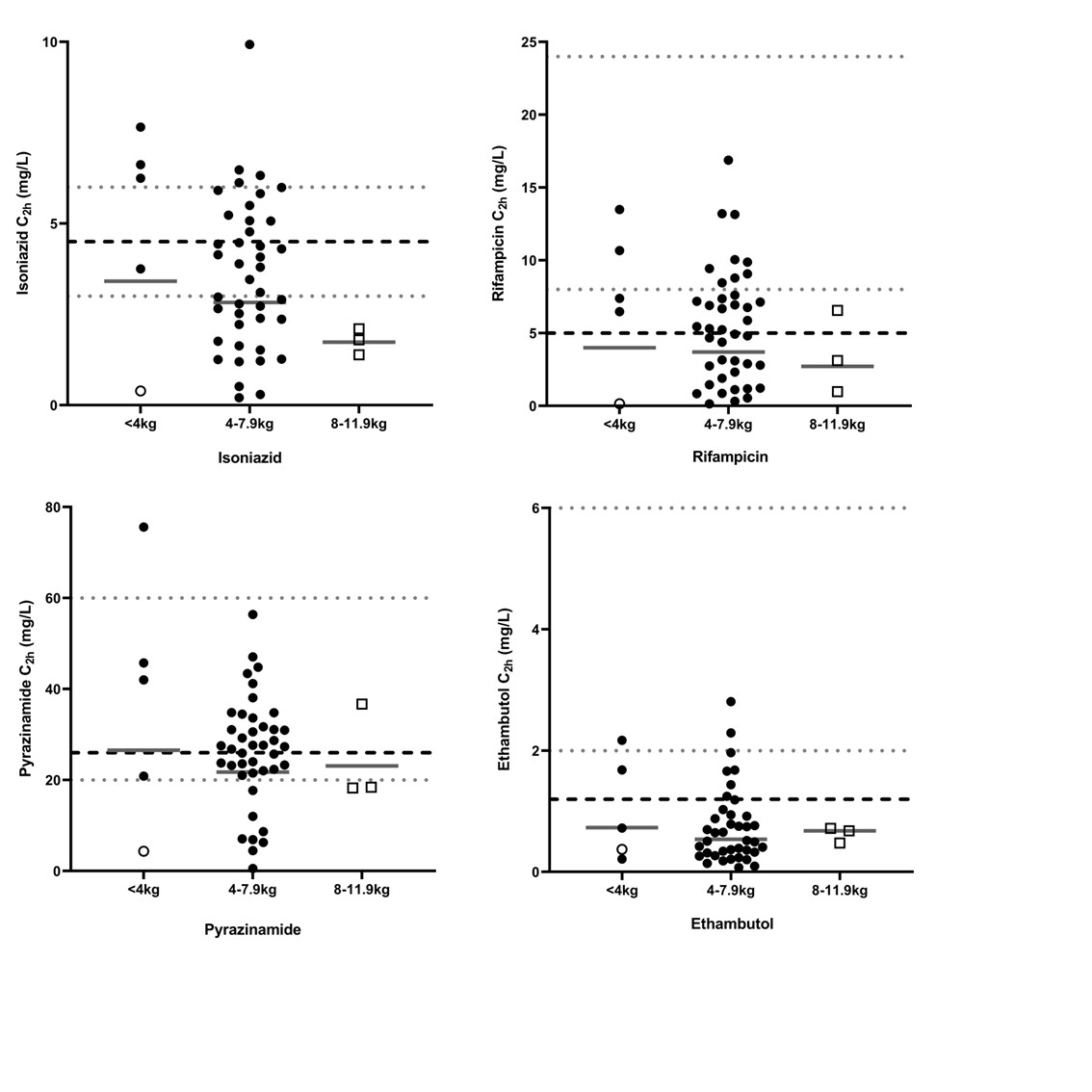


**Figure 2 Individual C_2h_ levels aggregated per weight-band.** The infant (weight <4kg) who has received 25mg isoniazid, 37.5mg rifampicin, 75mg pyrazinamide, and 50mg ethambutol is indicated a as **open dot**; Infants that have received 50mg isoniazid, 75mg rifampicin, 150mg pyrazinamide, and 100mg ethambutol are indicated as **black dots**; and those who have received 100mg isoniazid, 150mg rifampicin, 300mg pyrazinamide, and 200mg ethambutol are indicated as **open squares.** The grey solid line represents the geometric mean ratio of the individual C_2h_, the grey dotted line represent the adult target C_max_, and the black dashed line represents the median C_max_ for children within the 4-7.9kg weight-band as reported by Chabala et al. Top left panel: isoniazid; top right panel: rifampicin; bottom left panel: pyrazinamide; bottom right panel: ethambutol.

## Supplementary file 4 – Isoniazid and rifampicin C_2h_ at various study visits


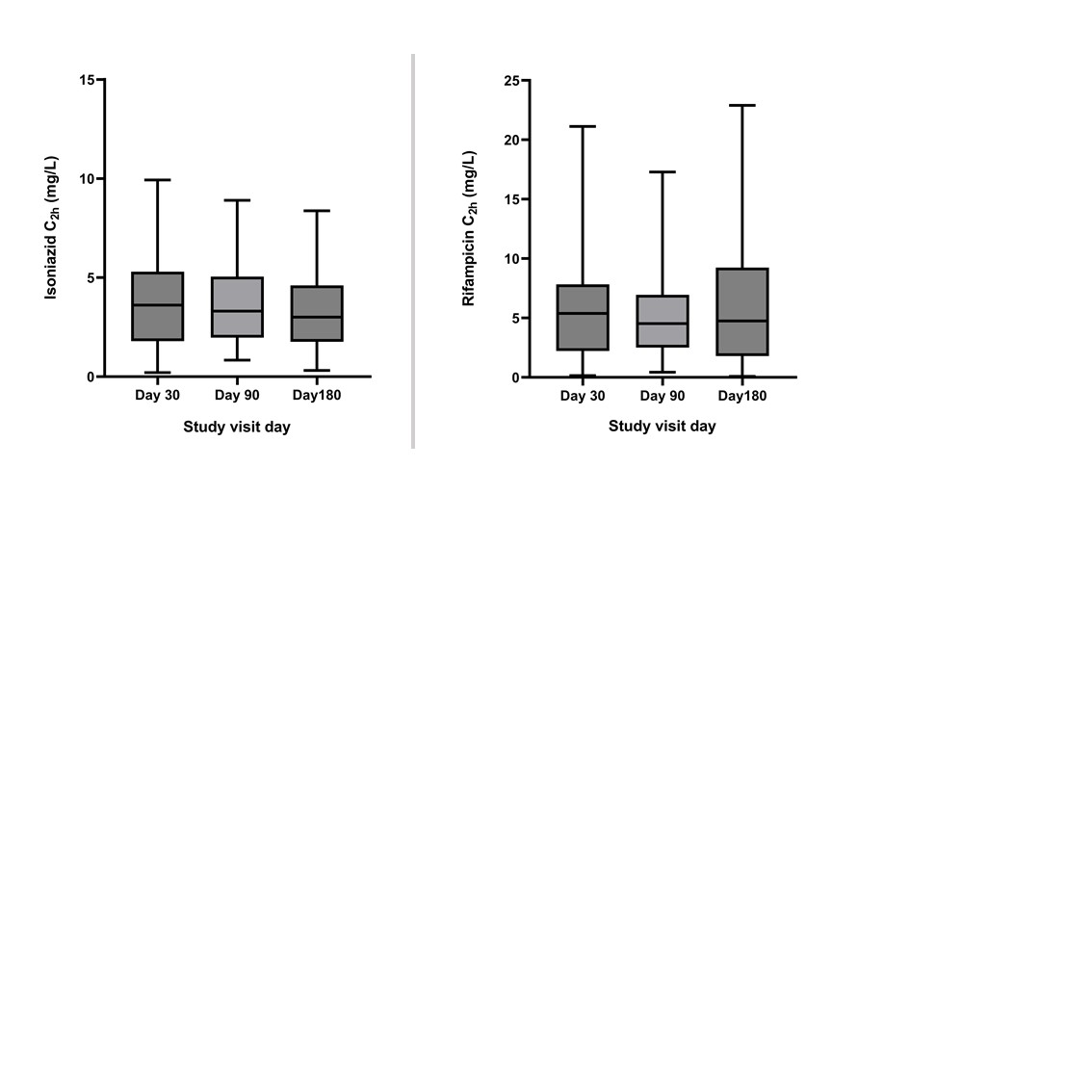


**Figure 3. Isoniazid and rifampicin C2h values over time.** Isoniazid (left panel) and rifampicin (right panel) plasma concentration at 2 hours after dose administration on visit day 30, 90, and 180 of the trial.

We used a one-way repeated measures ANOVA on log-transformed C_2h_ values to test for differences in C_2h_ between the various study visit days for isoniazid and rifampicin. Only infants with data available for all three visits were included (n = 29). No statistical difference in C_2h_ between the various study visit days was found for isoniazid (p = .865) and rifampicin (p = .971).

## Supplementary file 5 – Isoniazid C_2h_ aggregated per phenotypic acetylator status


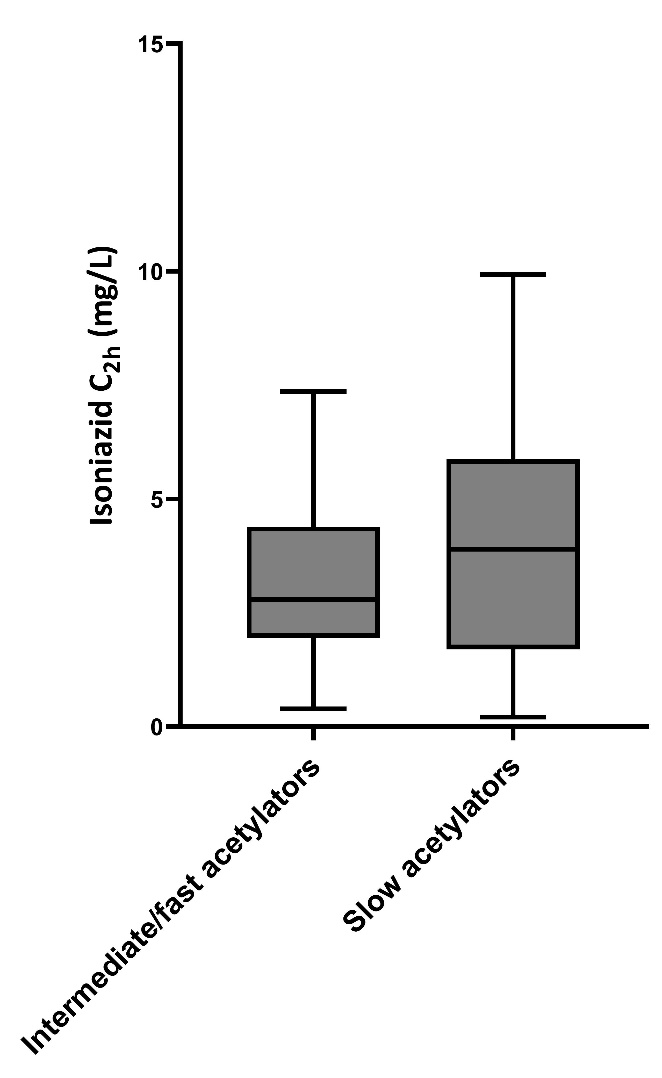


**Figure 4 Isoniazid C2h values for children who were considered slow metabolizers and those considered intermediate/fast metabolizers.** Phenotypic INH acetylator status was determined by calculating the metabolic ratio for C_2h_ (acetyl-INH / INH). Infants having a metabolic ratio below 0.73 were considered slow acetylators and those with higher ratios intermediate/fast acetylators(1).

## Supplementary file 6 – Results covariate analyses

| Covariate | Correlation coefficient (p-value) | | | |
| --- | --- | --- | --- | --- |
|  | *INH* | *RIF* | *PRZ* | *EMB* |
| Dose/kg | 0.314 (**.028**) | 0.330 (**.021**) | 0.354 (**.014**) | 0.268 (**.072**) |
| Height | -0.222 (.124) | -0.167 (.253) | -0.114 (.435) | -0.181 (.223) |
| WLZ | -0.420 (**.003**) | -0.349 (**.014**) | -0.432 (**.002**) | -0.047 (.752) |
| WAZ | -0.334 (**.019**) | -0.152 (.298) | -0.289 (**.044**) | -0.078 (.602) |
| Age | -0.235 (.104) | -0.298 (**.037**) | -0.085 (.560) | -0.081 (.587) |
| eGFR* | 0.187 (.208) | 0.006 (.969) | -0.062 (.681) | -0.472 (**.001**) |
|  | **Mann-Whitney z-value (p-value)** | | | |
| Sex | -1.475 (.140) | -0.364 (.716) | -0.121 (.904) | -2.796 (**.005**) |
| Acetylator status | -1.322 (.186) | - | - | - |

***Table 3*** *Spearman rank and Mann-Whitney U test results. Correlation between a selection of covariates and C_2h_ values of the TB drugs. All outcomes with p<.1 are highlighted in bolt as these were included in the multivariate analysis. p<.05 was considered statistically significant. Bold type: significant p-value
* mL/min; absolute eGFR not corrected for body surface area
Abbreviations: eGFR, estimated glomerular filtration rate; EMB, ethambutol; INH, isoniazid; PRZ, pyrazinamide; RIF, rifampicin; WAZ, weight-for-age z-score; WLZ, weight-for-length z-score.*

| Dependent variable | *Adjusted R^2^* | *F-value* | *p-value* |
| --- | --- | --- | --- |
| LN INH C_2h_ | 0.079 | 2.368 | .83 |
| LN RIF C_2h_ | 0.125 | 3.293 | **.029** |
| LN PRZ C_2h_ | 0.144 | 3.643 | **.020** |
| LN EMB C_2h_ | 0.250 | 5.785 | **.002** |

**Table 4** Multivariable analyses characteristics. All covariates with p<.1 in the univariate analyses were included in the multilinear regression model. Bold type: significant p-value
Abbreviations: EMB, ethambutol; INH, isoniazid; LN, natural logarithm; PRZ, pyrazinamide; RIF, rifampicin.

| Covariate | B-coefficient (p-value) | | | |
| --- | --- | --- | --- | --- |
|  | *INH* | *RIF* | *PRZ* | *EMB* |
| Dose/kg | 0.070 (.295) | 0.053 (.389) | 0.029 (.172) | 0.030 (.260) |
| Height |  |  |  |  |
| WLZ | -0.136 (.124) | -0.224 (**.040**) | -0.163 (.051) |  |
| WAZ | -0.030 (.754) |  | -0.013 (.885) |  |
| Age |  | -0.002 (.369) |  |  |
| eGFR |  |  |  | -0.010 (.100) |
| Sex |  |  |  | -0.584 (**.005**) |
| Acetylator status |  |  |  |  |

**Table 5** Contribution of individual covariates to the multilinear regression model. The dependent variable included the natural logarithm of C_2h_ values per drug. Bold type: significant p-value
Abbreviations: eGFR, estimated glomerular filtration rate; EMB, ethambutol; INH, isoniazid; PRZ, pyrazinamide; RIF, rifampicin; WAZ, weight-for-age z-score; WLZ, weight-for-length z-score.

## References

1. Verhagen LM, Coenen MJ, López D, García JF, de Waard JH, Schijvenaars MM, et al. Full-gene sequencing analysis of NAT2 and its relationship with isoniazid pharmacokinetics in Venezuelan children with tuberculosis. Pharmacogenomics. 2014;15(3):285-96.
